# Editorial: Sustainable synthesis for obtaining elements of natural origin with antimicrobial properties

**DOI:** 10.3389/fchem.2025.1664305

**Published:** 2025-07-22

**Authors:** Jose Vega-Baudrit, Yohann Corvis, Patricia Vázquez, Romina Andrae Arreche

**Affiliations:** ^1^ LANOTEC CENAT National Center for High Technology, San José, Costa Rica; ^2^Chemistry School, National University, Heredia, Costa Rica; ^3^ School of Pharmacy, Faculté of Santé, Université Paris Cité, Paris, France; ^4^ CINDECA (CONICET-CIC-UNLP), Universidad Nacional de la Plata, La Plata, Argentina

**Keywords:** synthesis, AMR (antimicrobial resistance), biogenic, material science, nanotechnology (drug discovery)

The escalation of antimicrobial resistance (AMR) poses an existential threat to global public health, underscoring a pressing need for novel strategies that not only effectively combat microbial pathogens but also uphold the principles of environmental sustainability. In this context, the Research Topic “Sustainable Synthesis for Obtaining Elements of Natural Origin with Antimicrobial Properties” converges on the promise of biogenic, green, and bio-inspired materials that offer potent antimicrobial effects while minimizing ecological impact. The four contributions to this Research Topic embody a multidisciplinary approach encompassing materials science, nanotechnology, microbial ecology, environmental chemistry, and pharmaceutical formulation, thus illuminating the intersection of natural systems and engineered solutions.

In their compelling contribution, Lopez et al. explore the sustainable functionalization of natural silica with citronellol and quaternary ammonium salts (QAS) to generate a novel hybrid antimicrobial material. The study centers around diatomaceous earth (Dt), a naturally occurring amorphous silica sourced from fossilized diatom microalgae. Its high porosity, low cost, and eco-friendliness make Dt an ideal platform for hybridization with biogenic agents. The authors demonstrate that Dt can be functionalized through a low-energy process, resulting in Dt*QC, a material that exhibits broad-spectrum antimicrobial activity. Structural characterization confirmed organic incorporation, and MIC values as low as 0.15 mg/mL were reported for *Staphylococcus aureus*.

Des Bouillons-Gamboa et al. focus on chitosan nanoparticles (CSNPs) produced via ionotropic gelation, optimizing the CS: TPP ratio to achieve colloidal stability and high antimicrobial activity. A 4:1 ratio provided nanoparticles with a size of ∼195 nm and a zeta potential of +51 mV. However, lyophilization caused aggregation and reduced efficacy, highlighting the need for cryoprotectants. These findings inform the development of chitosan-based delivery systems in antimicrobial therapy.

Chacón-Calderón et al. investigate the catalytic potential of mangrove soil extracts for the green synthesis of silver nanoparticles (AgNPs). Organic matter from Costa Rican mangrove sediments reduces silver ions under sunlight in 15 min, producing AgNPs with strong antimicrobial properties. Mechanistic studies using CV, UV-Vis, ITC, and ROS detection reveal the role of redox-active species and trace metals in nanoparticle formation. This research bridges environmental chemistry with the synthesis of functional materials. The Corrigendum by Arias-Chavarría et al. corrects affiliations in a study on *Trichoderma longibrachiatum* encapsulation. This work aligns with sustainable biocontrol strategies that utilize microencapsulation to extend the shelf life and efficacy of microbial agents.

These contributions reflect the growing recognition that nature not only inspires but enables the design of next-generation antimicrobial materials. Three thematic convergences emerge: redox-active natural systems that induce antimicrobial functions; surface functionalization and nanoarchitecture that modulate microbial interactions; and biocompatible and green chemistry methodologies aligned with ecological stewardship. Together, they offer pathways for scalable and eco-ethical innovation to confront antimicrobial resistance. We extend our gratitude to the authors and reviewers who made this Research Topic possible and trust that its findings will stimulate further interdisciplinary collaboration toward a more sustainable future in antimicrobial chemistry.

